# Long-Term Preservation of High-Frequency Hearing After Cochlear Implantation in a Child with Substantial Residual Hearing: A Case Report

**DOI:** 10.3390/jcm15208133

**Published:** 2026-10-20

**Authors:** Henryk Skarzynski, Elzbieta Wlodarczyk, Artur Lorens, Beata Dziendziel, Piotr H. Skarzynski

**Affiliations:** 1 Oto-Rhino-Laryngology Surgery Clinic, World Hearing Center, Institute of Physiology and Pathology of Hearing, 05-830 Kajetany, Poland; skarzynski.henryk@ifps.org.pl; 2 Rehabilitation Clinic, World Hearing Center, Institute of Physiology and Pathology of Hearing, 05-830 Warsaw, Poland; b.dziendziel@ifps.org.pl; 3 Implants and Auditory Perception Department, World Hearing Center, Institute of Physiology and Pathology of Hearing, 05-830 Kajetany, Poland; a.lorens@ifps.org.pl; 4 Teleaudiology and Screening Department, World Hearing Center, Institute of Physiology and Pathology of Hearing, 05-830 Kajetany, Poland; p.skarzynski@ifps.org.pl; 5 Heart Failure and Cardiac Rehabilitation Department, Medical University of Warsaw, 02-091 Warsaw, Poland; 6 Institute of Sensory Organs, 05-830 Kajetany, Poland

**Keywords:** cochlear implants, cochlear implantation, adolescent, hearing loss, sensorineural, speech perception, case reports

## Abstract

**Background/Objectives**: Cochlear implantation (CI) in children with substantial residual hearing remains challenging because of the risk of hearing loss. Partial deafness treatment and hearing preservation have traditionally focused on functionally useful low-frequency hearing, while residual hearing in the high-frequency range has not been specifically addressed in established classifications. Long-term data on preservation of substantial high-frequency residual hearing after CI in children remain limited. We present a pediatric case demonstrating 5-year preservation of high-frequency residual hearing after CI. **Methods**: A 14-year-old girl with bilateral sensorineural hearing loss underwent left CI with a MED-EL FLEX 28 electrode inserted through the round window. Pure-tone thresholds were assessed preoperatively and at 1, 12, 24, and 60 months postoperatively. Hearing preservation was evaluated using the Skarzynski classification, and speech perception was assessed before implantation and at 24 months. **Results**: Preoperatively, air-conduction thresholds were ≤70 dB HL at 2000–8000 Hz. Overall hearing preservation was complete at 1, 12, and 24 months and partial at 60 months. Importantly, hearing preservation at 2000–8000 Hz remained complete throughout the 60-month follow-up, with S-values of 90.7%, 76.7%, 79.1%, and 76.7%, respectively. At 24 months, word recognition in the implanted ear improved by 35 percentage points in quiet and 70 percentage points in noise compared with the preoperative aided condition. **Conclusions**: This case demonstrates 5-year preservation of substantial high-frequency residual hearing after CI in an adolescent with an atypical preoperative hearing profile. These findings suggest that cochlear implantation may be considered in selected patients with substantial residual hearing when conventional hearing aids provide insufficient functional benefit. However, broader conclusions regarding cochlear implant candidacy cannot be drawn from a single case.

## 1. Background

Pediatric cochlear implantation has evolved substantially since its introduction, with advances in surgical techniques, implant technology, and perioperative care progressively expanding candidacy criteria [1,2,3,4].

A major development was the introduction of cochlear implantation for patients with partial deafness. In 1999, von Ilberg et al. [5] introduced the concept of combined electric–acoustic stimulation, demonstrating the potential to combine preserved low-frequency acoustic hearing with electrical stimulation at higher frequencies. Skarzynski et al. [6] subsequently developed the first concept of Partial Deafness Treatment (PDT) including four audiological profiles: Electrical Complementation (EC), Electro-Natural Stimulation (ENS), Electro-Acoustic Stimulation (EAS), and Electrical Stimulation (ES). The EC, ENS, and EAS profiles are primarily characterized by functionally useful residual hearing in the low-frequency range, whereas ES refers to patients with non-functional residual hearing who rely exclusively on electrical stimulation [6]. A recent systematic review and meta-analysis including 24 studies and 567 pediatric patients reported hearing preservation in 78% of implanted ears (95% CI: 71–85%), although substantial heterogeneity was observed across studies [7].

In patients with partial deafness, preservation of preoperative residual hearing has become an important goal of cochlear implantation, allowing acoustic hearing to complement electrical stimulation and potentially contributing to improved auditory outcomes [6]. Hearing preservation is also considered an indicator of reduced intracochlear trauma during electrode insertion [8]. Minimizing intracochlear damage may reduce inflammatory responses, fibrosis, and neo-ossification, which may be relevant for long-term cochlear function and potential future interventions [9,10,11].

The prevalence and clinical characteristics of partial deafness in children remain incompletely defined [12]. In children with substantial residual hearing, cochlear implant candidacy can be particularly challenging because the potential auditory benefit must be balanced against the risk of losing residual hearing. A systematic review by Na et al. [13] reported improvements in speech perception following cochlear implantation in children with residual hearing, despite postoperative hearing loss in some patients. Furthermore, a recent prospective study by Park et al. [14] demonstrated meaningful auditory benefits following cochlear implantation in children with functional preoperative residual hearing. Importantly, limited benefit from conventional hearing aids may not always be adequately reflected by pure-tone thresholds alone, potentially delaying referral for cochlear implant evaluation [15,16].

Although hearing preservation after cochlear implantation in children has been increasingly reported, the concept of partial deafness and the available pediatric literature have primarily focused on preservation of functionally useful low-frequency hearing [12,16]. Residual hearing extending into the high-frequency range has not been specifically addressed in established PDT classifications. A recent case series described cochlear implantation in patients with substantial residual hearing extending into the high-frequency range; however, these data concerned adult patients [17]. Long-term data on the preservation of substantial high-frequency residual hearing after cochlear implantation in children remain limited.

In this case report, we present a 14-year-old patient with an atypical residual-hearing profile, characterized by substantial hearing across the low-, mid-, and high-frequency ranges, including functional hearing at 2000–8000 Hz, despite limited benefit from conventional hearing aids. Cochlear implantation was performed despite the presence of substantial high-frequency residual hearing. Complete preservation of hearing in the 2000–8000 Hz range was maintained throughout 60 months of follow-up, accompanied by substantial improvement in speech perception, particularly in noise.

The aim of this case report is to describe long-term preservation of substantial high-frequency residual hearing and speech perception outcomes following cochlear implantation in an adolescent with an atypical preoperative hearing profile.

## 2. Case Presentation

This case report was prepared with reference to the CARE reporting guidelines, with most of the applicable checklist items addressed in the manuscript. The completed CARE checklist, including items that were not reported, is provided as a Supplementary Materials. A 14-year-old girl underwent cochlear implantation in the left ear in March 2020 because of progressive bilateral sensorineural hearing loss and limited benefit from conventional hearing aids. Before cochlear implantation, the patient retained measurable hearing across the entire tested frequency range. Air-conduction thresholds did not exceed 80 dB HL between 125 and 1500 Hz, whereas values of no more than 70 dB HL were recorded at frequencies from 2000 to 8000 Hz.

Bilateral sensorineural hearing loss was identified shortly after birth and demonstrated a progressive course over subsequent years. From the age of 6 months, the patient used bilateral conventional air-conduction hearing aids and participated regularly in speech therapy. Regular hearing-aid use and speech therapy supported her speech development, and she attended a mainstream primary school. Genetic testing confirmed the presence of the 35delG variant in both copies of the *GJB2* gene, indicating that the patient’s hearing loss was primarily of genetic origin. Serological testing performed at 6 months of age also showed elevated antibody titers against cytomegalovirus (CMV). However, the medical records did not specify the antibody class or provide information on confirmatory testing. Therefore, congenital CMV infection could not be confirmed based on the available documentation.

The first diagnostic evaluation for cochlear implantation was performed at the age of 12 years. Based on the results of audiological assessment, including pure-tone audiometry and speech perception testing, the patient did not meet the criteria for cochlear implantation at that time, and implantation was deferred. Two years later, a repeat audiological evaluation was performed because of increasing communication difficulties, particularly in the school environment, as reported by the patient. Although pure-tone audiometry continued to demonstrate substantial residual hearing in the high-frequency range, aided speech recognition in quiet in the left ear was 80% at the earlier assessment and 55% at the later assessment. The patient was also approaching the transition to the next stage of education, providing additional context for the reported communication difficulties. Based on the audiological findings, and insufficient functional benefit from conventional hearing-aid amplification, the left ear was selected for cochlear implantation. Given the patient’s substantial preoperative residual hearing, both the patient and the parents were informed about the risk of deterioration or loss of residual hearing, including hearing in the high-frequency range, during the cochlear implant candidacy evaluation. This risk was discussed again during the preoperative consultation, and written informed consent for cochlear implantation was obtained. The overall clinical course is summarized in Table 1.

### 2.1. Preoperative Audiometric Evaluation

**Figure 1:**
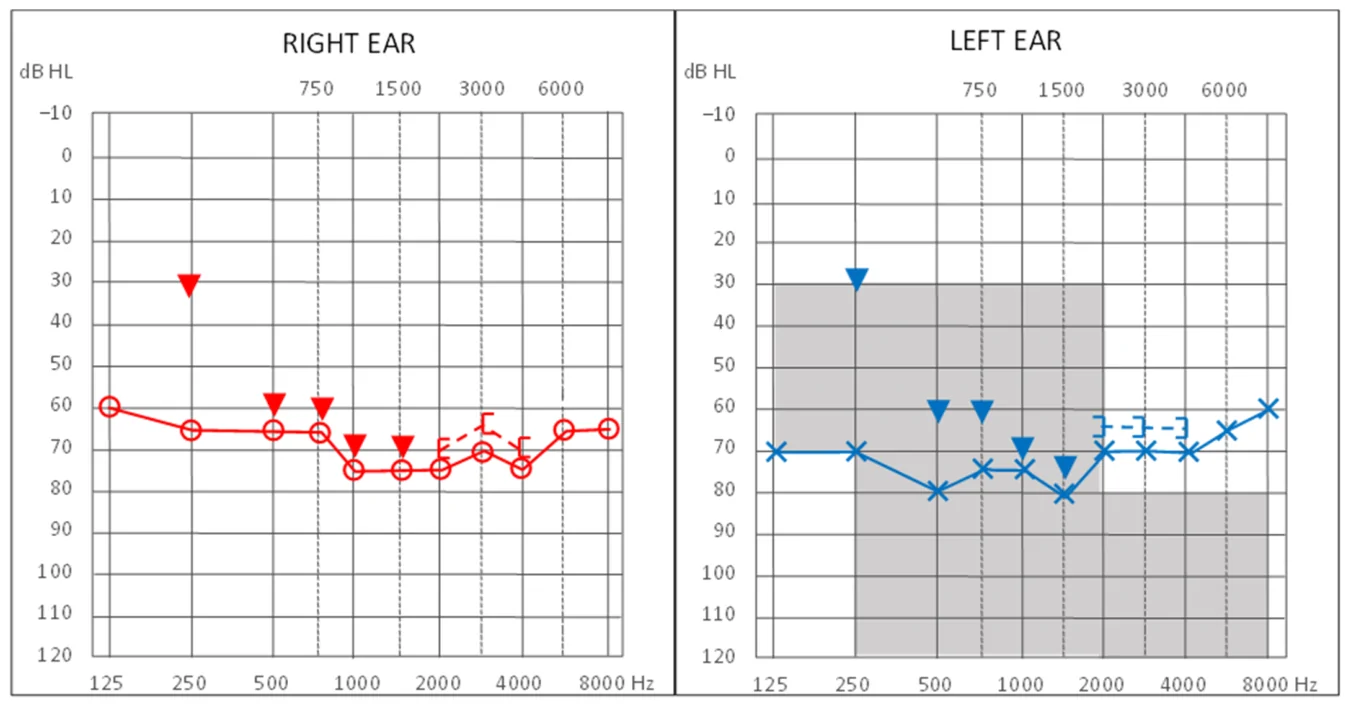
Preoperative pure-tone audiometry. Shaded area indicates electro-acoustic stimulation criteria according to Skarzynski’s concept.

Preoperative hearing assessment at the age of 14 included pure-tone audiometry, with air-conduction (AC) thresholds measured from 125 to 8000 Hz and bone-conduction (BC) thresholds from 250 to 4000 Hz, in accordance with ISO 8253-1:2010 [18]. Unlike the predominantly low-frequency residual hearing typically observed in candidates for hearing preservation cochlear implantation, this patient exhibited an atypical audiometric profile, with residual hearing extending into the high-frequency range (2000–8000 Hz). Figure 1 illustrates the preoperative audiometric findings. The four-frequency pure-tone average (PTA), derived from thresholds at 500, 1000, 2000, and 4000 Hz, was 73.8 dB HL in the right ear and 72.5 dB HL in the left ear.

### 2.2. Surgical Procedure

Because the preoperative low-frequency hearing thresholds (125–500 Hz) were at the borderline of functional hearing and were considered insufficient to provide a meaningful acoustic component, an exclusively electrical stimulation strategy was selected. To minimize intracochlear trauma, a flexible MED-EL FLEX 28 electrode (MED-EL GmbH, Innsbruck, Austria) was used. Postoperative computed tomography (CT) confirmed an angular insertion depth of approximately 590°, with the electrode array extending through the basal and middle turns of the cochlea and all electrode contacts located within the cochlea. The electrode array was inserted via the round window using the six-step surgical technique described by Skarzynski et al. [6,19]. The procedure comprised the following stages:

Step 1: A conservative antromastoidotomy was performed, and a bone chip was harvested from the mastoid cortex for subsequent closure of the middle ear space.

Step 2: A limited posterior tympanotomy was performed to expose the round window.

Step 3: The round window membrane was punctured and incised centrally.

Step 4: The flexible Synchrony FLEX 28 electrode array was advanced into the scala tympani at an appropriate insertion angle.

Step 5: The electrode array was stabilized in the round window niche using tissue glue and a piece of fascia.

Step 6: The internal implant component was positioned in a surgically prepared bony well. The mastoid cavity was then closed using the previously harvested bone chip, followed by wound suturing.

Perioperative steroid therapy consisted of intravenous dexamethasone administered at a dose of 0.1 mg/kg body weight, beginning 30 min before cochlear implantation. The same dose was subsequently administered intravenously every 12 h for three consecutive days. Following this treatment, at hospital discharge, the patient was prescribed oral prednisone, initially at a dose of 40 mg daily for five days, followed by a gradual dose reduction of 5 mg per day until discontinuation.

### 2.3. Postoperative Audiometric Evaluation

**Figure 2:**
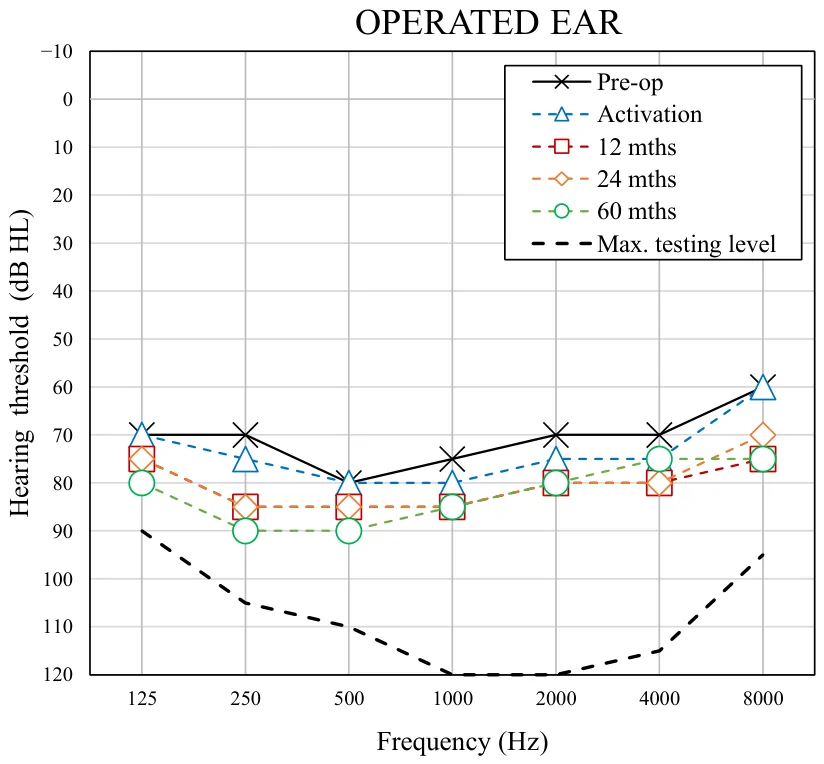
Preoperative and postoperative air-conduction hearing thresholds in the implanted ear.

Postoperative pure-tone audiometry was conducted 1 month after surgery, at implant activation, at implant activation, and repeated at 12, 24, and 60 months. Figure 2 shows the preoperative and postoperative AC thresholds in the implanted ear.

The mean preoperative AC threshold across all tested frequencies was 71.4 dB HL, increasing by 3.2 dB HL at implant activation. At the subsequent follow-up visits (12, 24, and 60 months), mean AC thresholds showed relatively stable, with a maximum threshold shift of 11 dB HL relative to baseline. relative to baseline. For the high-frequency range (2000, 3000, 4000, 6000, and 8000 Hz), the preoperative mean AC threshold was 67 dB HL. This value increased by 4 dB HL at activation and remained relatively stable thereafter, with a maximum threshold shift of 10 dB HL compared with preoperative values. Both preoperatively and postoperatively, BC thresholds were measurable only at selected high frequencies (2000–4000 Hz), while responses at lower frequencies (250–1500 Hz) were perceived as vibrations rather than sound, partly due to the audiometer’s maximum output limitations. Consequently, the limited BC data precluded longitudinal analysis.

### 2.4. Hearing Preservation Evaluation

Hearing preservation (HP) was assessed according to the classification system developed by Skarzynski et al. [20], which considers changes in pure-tone hearing thresholds between preoperative and postoperative measurements across 11 audiometric frequencies (0.125, 0.25, 0.5, 0.75, 1, 1.5, 2, 3, 4, 6, and 8 kHz). The degree of HP was calculated using the formula:

$$S=\left(1-\frac{\mathrm{PTApost}-\mathrm{PTApre}}{\mathrm{PTAmax}-\mathrm{PTApre}}\right)*100\;[\%]$$

In this equation, PTApre is the pure tone-average measured preoperatively, PTApost is the pure tone average measured postoperatively, and PTAmax is the maximum level generated by a standard audiometer [20]. Based on the S-values, HP can be stratified into four categories: loss of hearing (no detectable hearing), minimal HP (range 1–25%), partial HP (26–75%), and complete HP (>75%). This formula allows for the quantitative assessment of overall postoperative hearing preservation based on changes in pure-tone hearing thresholds. Hearing thresholds were assessed at five time points: preoperatively, at activation visit, and at 12, 24, and 60 months after surgery. The resulting S-values are presented in Table 2. According to the HP classification, complete hearing preservation was observed at 1, 12, and 24 months after surgery, with S-values of 91.8%, 77.8%, and 79.0%, respectively. At 60 months, the S-value was 73.1%, corresponding to partial hearing preservation according to the classification criteria.

Given the patient’s atypical preoperative hearing profile, hearing preservation was additionally assessed in the high-frequency range (2000, 3000, 4000, 6000, and 8000 Hz) using the same formula, with PTA values calculated exclusively from these five frequencies. The calculated S-values were 90.7% at 1 month, 76.7% at 12 months, 79.1% at 24 months, and 76.7% at 60 months after surgery. According to the same classification, these values corresponded to complete hearing preservation in the high-frequency range throughout the entire 60-month follow-up period. Thus, although overall hearing preservation was classified as partial at 60 months, hearing preservation within the high-frequency range remained complete according to the same classification criteria.

### 2.5. Speech Discrimination Evaluation

Preoperative speech audiometry was conducted with the Demenko and Pruszewicz Polish Monosyllabic Word Test [21]. The maximum presentation level available on the standard audiometer was 100 dB SPL (sound pressure level). The speech detection threshold (SDT) was 85 dB SPL in both ears. With the presentation level set to its maximum of 100 dB SPL, the maximum speech recognition score (Smax) reached 40% in the right ear and 35% in the left ear. The patient did not achieve the speech recognition threshold (SRT), corresponding to the minimum presentation level required for correct repetition or identification of 50% of the presented speech material.

**Figure 3:**
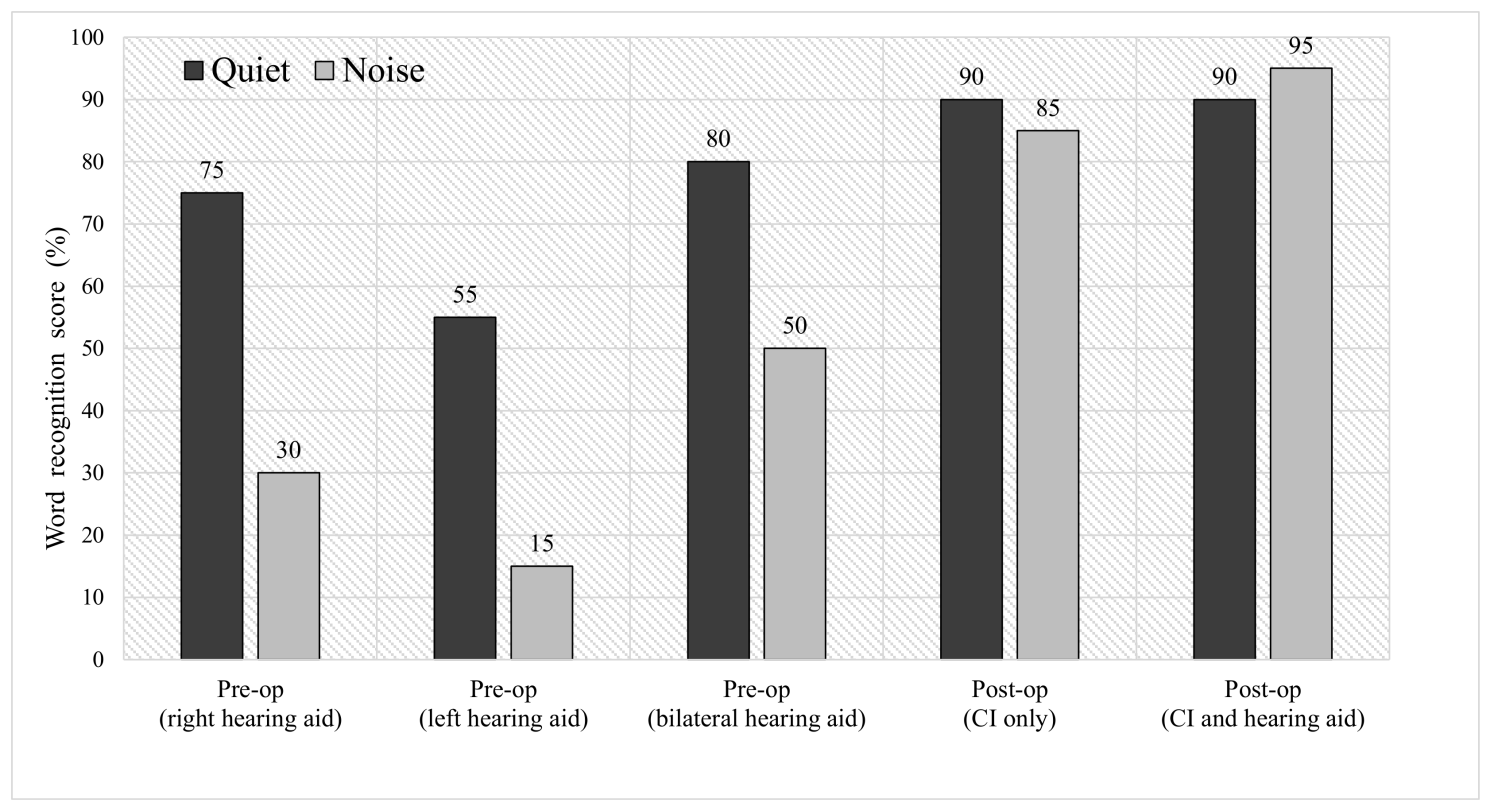
Free-field word recognition score in quiet and noise measured preoperatively and 24 months following cochlear implantation.

The same Demenko and Pruszewicz Polish Monosyllabic Word Test [21] was used to assess word recognition in the free field before and after cochlear implantation. Preoperative testing was performed in unaided and aided conditions using the patient’s conventional hearing aids. Postoperative testing was performed following cochlear implant activation to assess the auditory benefit provided by the CI. Speech perception was evaluated at 65 dB SPL under both quiet and noise conditions, with CCITT speech-shaped noise presented at a signal-to-noise ratio of +10 dB. A loudspeaker was placed 1 m directly in front of the patient (S0 azimuth). The contralateral ear was occluded. Before implantation, the word recognition score (WRS) was 0% in quiet for both ears without hearing aids. After 24 months of exclusive CI use in the implanted left ear, WRS increased by 35 percentage points in quiet and by 70 percentage points in noise relative to the preoperative aided condition. To additionally assess the change in overall aided speech perception, the preoperative best-aided condition (bilateral hearing aids) was compared with the postoperative best-aided condition (CI in the left ear and hearing aid in the right ear). An increase in WRS of 10 percentage points was observed in quiet, compared with an improvement of 45 percentage points in noise. The WRS results obtained in the free field before cochlear implantation and at 24 months after implantation, in quiet and noise conditions, are presented in Figure 3.

## 3. Discussion

Cochlear implantation is an established treatment for children with severe-to-profound hearing loss; however, its application in those with substantial residual hearing continues to pose clinical challenges. In such cases, the potential benefit of cochlear implantation must be carefully balanced against the risk of losing residual acoustic hearing. Determining whether conventional hearing aids provide sufficient benefit may further delay referral for cochlear implant evaluation [16].

At our center, a pediatric cochlear implantation program for patients with partial deafness has been implemented since 2004 [6]. The patient described in this report presented a particularly challenging candidacy profile because, despite substantial residual hearing across the low-, mid-, and high-frequency ranges, conventional hearing aids provided insufficient speech perception, particularly in challenging listening environments. Her audiometric profile differed from the typical PDT patterns, in which functionally useful residual hearing is predominantly preserved in the low-frequency range, as functional residual hearing in this case extended to 2000–8000 Hz.

The discrepancy between pure-tone thresholds and speech perception was an important factor in the decision-making process. Preoperatively, the patient had substantial residual hearing at high frequencies but demonstrated limited speech recognition with hearing-aid amplification. Pure-tone audiometry does not necessarily reflect the functional status of the cochlea or the quality of speech perception [22]. One possible explanation for poor speech perception despite measurable residual hearing is the presence of cochlear dead regions, which may limit the benefit obtained from amplification [23,24,25,26,27,28]. However, dead regions were not specifically assessed in the present patient, and their possible presence therefore remains speculative. Nevertheless, this case illustrates that audiometric thresholds alone may not fully characterize the functional benefit obtained from hearing aids and that speech perception should be considered when evaluating borderline cochlear implant candidates.

The patient had congenital, progressive sensorineural hearing loss associated with a homozygous 35delG mutation in the *GJB2* gene. The *GJB2* gene encodes connexin 26, which plays an important role in cochlear function. Studies by Ross et al. [29] and Li et al. [30] have examined the relationship between congenital CMV infection and *GJB2* mutations, with differing findings that do not establish a causal or synergistic interaction between these factors. In this patient, the homozygous 35delG mutation supports a primarily genetic etiology, whereas congenital CMV infection could not be confirmed based on the available medical records, leaving a possible additional contribution of CMV uncertain. Previous studies have shown that *GJB2*-related deafness is associated with favorable cochlear implantation outcomes compared with some acquired forms of sensorineural hearing loss [31,32,33]. In the present case, the genetic etiology may therefore have contributed to the favorable postoperative auditory outcome. However, the contribution of the genetic etiology to hearing preservation cannot be determined from a single case.

A flexible MED-EL FLEX 28 electrode was selected, and the electrode was inserted through the round window using a surgical technique designed to minimize intracochlear trauma. Because the patient’s low-frequency residual hearing was considered at the borderline of functional usefulness, an EAS strategy was not considered appropriate in this case, particularly from a long-term perspective. Previous studies have suggested that electrode insertion depth may influence speech perception outcomes [34,35]. At the same time, deeper electrode insertion may raise concerns regarding preservation of residual hearing. In the present case, complete hearing preservation according to the applied classification was observed up to 24 months after implantation, while partial hearing preservation was observed at 60 months when all tested frequencies were considered. However, the S-value at 60 months (73.1%) was only slightly below the 75% cutoff for complete hearing preservation. This marginal difference reflects the categorical nature of the classification and does not necessarily indicate a clinically meaningful deterioration in residual hearing. Importantly, when the high-frequency range was analyzed separately, complete hearing preservation was maintained throughout the entire 60-month follow-up period. This finding is particularly relevant because the patient’s preoperative residual hearing was not limited to the low-frequency range but remained functionally useful at 2000–8000 Hz. Long-term preservation of residual hearing has also been reported in pediatric cochlear implant recipients, although hearing thresholds may deteriorate over time [36,37]. However, direct comparison with these studies is limited by differences in hearing preservation assessment methods and the predominantly low-frequency focus of previous research. The present findings suggest that high-frequency residual hearing may also be preserved over the long term in selected pediatric patients, although this observation requires confirmation in larger studies. Nevertheless, hearing preservation alone cannot be used to directly determine the absence of intracochlear trauma. The extent of hearing preservation is influenced by multiple factors, including electrode design, cochlear anatomy, insertion angle, insertion depth, and surgical technique [38]. The round window approach has also been associated with preservation of cochlear structures and may reduce the risk of trauma and subsequent fibrosis [39,40].

The postoperative speech perception results provide an additional clinically relevant finding. At 24 months after implantation, speech recognition in the operated ear improved substantially compared with the preoperative aided condition, with greater improvement observed in noise than in quiet. The improvement was also observed when comparing the preoperative best-aided condition (bilateral hearing aids) with the postoperative bimodal condition (cochlear implant in the left ear and hearing aid in the right ear). These findings indicate that cochlear implantation provided additional functional benefit despite the preservation of substantial preoperative residual hearing. The present case differs from previously described adult patients with similar audiometric configurations [17]. In those patients, preoperative speech perception with hearing aids was generally poor despite measurable residual hearing. In the present pediatric patient, preoperative speech perception was comparatively better, but it was nevertheless insufficient for effective communication in everyday environments, particularly at school. The favorable postoperative outcome suggests that the decision to implant should not necessarily be based on pure-tone thresholds alone when conventional amplification provides limited functional benefit.

This case has several limitations. First, it represents a single pediatric case and therefore cannot establish the frequency or predictability of high-frequency hearing preservation after cochlear implantation. Second, the case was evaluated retrospectively, and cochlear dead regions were not assessed preoperatively. Third, the findings cannot establish a causal relationship between the surgical technique, electrode design, and preservation of high-frequency hearing. Nevertheless, the 60-month follow-up provides clinically relevant long-term information on a pediatric patient with an atypical residual-hearing profile that extends into the high-frequency range.

## 4. Conclusions

This case demonstrates that long-term preservation of substantial high-frequency residual hearing is possible following cochlear implantation in an adolescent with an atypical preoperative hearing profile. The findings suggest that cochlear implantation may be considered in selected patients with substantial residual hearing when conventional hearing aids provide insufficient functional benefit. However, conclusions regarding broader candidacy criteria cannot be drawn from a single case, and further studies are needed to confirm these observations.

## Figures and Tables

**Table 1:** Clinical timeline.

Time Point	Clinical Course	Key Findings or Actions
Shortly after birth	Diagnosis of hearing loss	Bilateral sensorineural hearing loss identified.
Age 6 months	Hearing aid fitting and initiation of speech therapy	Bilateral conventional hearing aids fitted; regular speech therapy initiated.
Age 12 years	First diagnostic evaluation for cochlear implantation	The decision regarding cochlear implantation was deferred.
Age 14 years	Second diagnostic evaluation for cochlear implantation	Increasing communication difficulties, particularly at school, and insufficient benefit from conventional hearing aids despite substantial residual hearing.
Age 14 years	Cochlear implantation procedure	Left cochlear implantation using a MED-EL FLEX 28 electrode (MED-EL GmbH, Innsbruck, Austria).
1 month postoperatively	Implant activation	Pure-tone audiometry and hearing preservation assessment.
12 months postoperatively	Audiological follow-up	Pure-tone audiometry and hearing preservation assessment.
24 months postoperatively	Audiological follow-up	Pure-tone audiometry, hearing preservation assessment, and speech perception testing.
60 months postoperatively	Long-term audiological follow-up	Pure-tone audiometry and hearing preservation assessment.

**Table 2:** Results of hearing preservation according to Skarzynski’s HP classification.

Postoperative Follow-Up	Hearing Preservation in (%)	Evaluation
1 month	91.8	Complete HP
12 months	77.8	Complete HP
24 months	79	Complete HP
60 months	73.1	Partial HP
Legend:		
S	Evaluation	
>75%	Complete HP	
26–75%	Partial HP	
1–25%	Minimal HP	
No detectable hearing	Loss of hearing	

Abbreviations: HP, hearing preservation; S, hearing preservation score.

## Data Availability

The datasets generated and/or analysed during the current study are not publicly available due to privacy and confidentiality considerations related to the individual patient but are available from the corresponding author on reasonable request and subject to applicable data protection and ethical requirements.

## Supplementary Materials

The following supporting information can be downloaded at: https://www.mdpi.com/article/10.3390/jcm15208133/s1, Table S1: CARE Checklist for a Case Report.

## Abbreviations

AC	air conduction
BC	bone conduction
CI	cochlear implant
Cx26	connexin 26
EAS	electro-acoustic stimulation
EC	electrical complementation
ENS	electro-natural stimulation
ES	electrical stimulation
HA	hearing aid
HP	hearing preservation
PDT	Partial Deafness Treatment
PTA	pure-tone average
SDT	speech detection threshold
Smax	maximum speech recognition score
SRT	speech recognition threshold
WRS	word recognition score
