# Supplementary material for: Long-Term Preservation of High-Frequency Hearing After Cochlear Implantation in a Child with Substantial Residual Hearing: A Case Report
Source: J Clin Med. 2026 Oct 20;15(20):8133. doi: 10.3390/jcm15208133 (PMC13705674; doi:10.3390/jcm15208133)
Supplement: Supplementary file 1 [file jcm-15-08133-s001.zip › jcm-4610374-supplementary.pdf]

CARE
case report guidelines

CARE Checklist of information to include when writing a case report

(cc) BY-NC-ND

Topic	Item	Checklist item description	Reported on Line
**Title**	1	The diagnosis or intervention of primary focus followed by the words "case report"	lines 1-4
**Key Words**	2	2 to 5 key words that identify diagnoses or interventions in this case report, including "case report"	lines 36-38
**Abstract (no references)**	3a	Introduction: What is unique about this case and what does it add to the scientific literature?	lines 13-20
	3b	Main symptoms and/or important clinical findings	lines 14-22
	3c	The main diagnoses, therapeutic interventions, and outcomes	lines 21-22, 25-31
	3d	Conclusion—What is the main "take-away" lesson(s) from this case?	lines 31-35
**Introduction**	4	One or two paragraphs summarizing why this case is unique (**may include references**)	line 66-84
**Patient Information**	5a	De-identified patient specific information.	lines 85-99
	5b	Primary concerns and symptoms of the patient.	lines 92-110
	5c	Medical, family, and psycho-social history including relevant genetic information	Genetic info: 96-98; Family history: not reported
	5d	Relevant past interventions with outcomes	lines 100-110
**Clinical Findings**	6	Describe significant physical examination (PE) and important clinical findings.	lines 111-117; 139-197
**Timeline**	7	Historical and current information from this episode of care organized as a timeline	lines 85-110; No separate timeline is provided.
**Diagnostic Assessment**	8a	Diagnostic testing (such as PE, laboratory testing, imaging, surveys).	lines 111-118; 145-197
	8b	Diagnostic challenges (such as access to testing, financial, or cultural)	lines 101-108
	8c	Diagnosis (including other diagnoses considered)	lines 86-99
	8d	Prognosis (such as staging in oncology) where applicable	Not reported
**Therapeutic Intervention**	9a	Types of therapeutic intervention (such as pharmacologic, surgical, preventive, self-care)	lines 92-96; 100-101; 121-138
	9b	Administration of therapeutic intervention (such as dosage, strength, duration)	lines 95-96; 121-138
	9c	Changes in therapeutic intervention (with rationale)	lines 121-138
**Follow-up and Outcomes**	10a	Clinician and patient-assessed outcomes (if available)	lines 139-197
	10b	Important follow-up diagnostic and other test results	lines 139-197
	10c	Intervention adherence and tolerability (How was this assessed?)	Not specifically reported
	10d	Adverse and unanticipated events	Not reported in the manuscript.
**Discussion**	11a	A scientific discussion of the strengths AND limitations associated with this case report	lines 275-282
	11b	Discussion of the relevant medical literature **with references**.	lines 203-274
	11c	The scientific rationale for any conclusions (including assessment of possible causes)	lines 217-274
	11d	The primary "take-away" lessons of this case report (without references) in a one paragraph conclusion	lines 283-293
**Patient Perspective**	12	The patient should share their perspective in one to two paragraphs on the treatment(s) they received	Not reported.
**Informed Consent**	13	Did the patient give informed consent? Please provide if requested	Yes ☐ No ☒

Further information: Informed Consent Statement (lines 308–311).
